# Application of pediatric index of mortality version 2: score in pediatric intensive care unit in an African developing country

**DOI:** 10.11604/pamj.2014.17.185.2818

**Published:** 2014-03-11

**Authors:** Osama El Sayed Mohamed Bekhit, AlKassem Ahmed Algameel, Hanaa Hasan Eldash

**Affiliations:** 1 Pediatrics Department, Faculty of Medicine, Al Fayoum University, Al Fayoum, Egypt

**Keywords:** PICU, PIM2, developing country

## Abstract

**Introduction:**

Outcome of patients admitted to PICU can be evaluated by many illness severity scoring systems. This prospective observational study evaluated the outcome of patients admitted to PICU in Fayoum University hospital of a developing country using the pediatric index of mortality version 2 scoring system.

**Methods:**

All patients included in this study were subjected to data collection including demographics, diagnoses at admission, duration of ICU stay (DOS), pediatric index of mortality version 2 (PIM2) score and hospital outcome. The ratio of observed to predicted mortality (standardized mortality ratio (SMR)) was calculated for the set of patients.

**Results:**

The study included 205 patients. The main causes of admission were respiratory, cardiovascular and neurological illnesses. Patients stay in ICU ranged from 1 - 45 days with a median 6 (interquartile range (IQ): 3-9) days. Discriminatory function of PIM2 scoring system was acceptable with the area under the ROC curve 0.76 (95%CI: 0.60-0.91). PIM2 calibrated well using Hosmer Lemeshow analysis (H-L X2= 1.410, df= 8, p=0.9). The mean predicted mortality was 5.6 (95% CI: 3.43 - 7.91) and the observed mortality was 8.8% giving a SMR 1.55.

**Conclusion:**

PIM2 scoring system show adequate discriminatory function and well calibrated for the case mix of patients in PICU of Fayoum, Egypt. It can be used as beneficial tool for evaluation of risk adjusted mortality. Further larger scale studies in cooperation with other Egyptian universities and neighboring countries can improve the performance of our PICUs and critical care services.

## Introduction

Mortality reduction is the fundamental aim of a pediatric intensive care unit (PICU). A physician's accuracy in estimating mortality risk for patients admitted to intensive care unit is largely subjective [1]. Scoring systems are used to predict the outcome of patients admitted to intensive care units [2]. Moreover they are used to evaluate the performance of ICUs [3]. Pediatric risk of mortality III is one of the principal scores used in PICUs throughout the world [4]. Because of the large differences in case mix, comparing the mortality among different units and countries must be corrected for the severity of illness on admission to the ICU and severity scoring systems have proved valuable for quality assurance and research in intensive care medicine [5].

Pediatric Index of Mortality 2 scoring system is the updated version of PIM introduced by Shann et al in 1997, with better outcome predictability. The score analyze the condition of the patient directly upon arrival in PICU, i.e the condition the least affected by any therapeutic interventions. This differs from other scoring systems, which could be applied at different time intervals throughout the ICU stay [6]. Comparison of intensive care outcomes at different regions is important because the employment of resources varies considerably in different countries especially the developing ones [7]. Pediatric index of mortality version 2 (PIM2) scoring system was validated in different regions including developed and developing countries, however reports about its application in our region are lacking.

The objective of this prospective study was to determine the performance of PIM2 score in Fayoum University Hospital pediatric ICU, to assess the quality of critical care services in our unit and to compare it with the international reports.

## Methods

Fayoum university Hospital is a tertiary care center that serves Fayoum Governorate with a 2000000 population. Pediatric ICU is a four bed unit. A resident and a senior registrar are in duty around the clock under supervision of a consultant. The nurse patient ratio is 1:1. The unit receives cases from general ward and emergency department including different medical specialties.

The ethical committee of Faculty of Medicine, Fayoum University approved the study. All consecutive admissions from May 2010 to June 2011 were included. Patients with a PICU stay less than 2 hours and those transferred to other PICU are excluded from the study.

Age, gender, diagnosis at the time of admission, duration of PICU stay and outcome (survived / death) were recorded in a data collection form devised for the study. PIM2 scoring system was applied on the day of admission before any therapeutic intervention was undertaken. The regression equation published with PIM2 scoring system was used to calculate the predicted mortality [6].

Data was summarized using mean and standard deviation or median and percentiles for quantitative variables and frequency and percentage for qualitative variables. Comparison between groups was done using Mann Whitney U-test for quantitative variables and chi square test or Fisher's exact test for qualitative variables.

Hosmer-Lemeshow goodness-of-fit analysis was done to calibrate the scoring system. Receiver Operating Characteristic curve (ROC) analysis was done to analyze the discriminant function of the system. Standardized Mortality Ratio (SMR) (the ratio of observed mortality to the predicted mortality) was obtained for the case mix. The statistical significance was fixed at p<0.05 level. Statistical analyses were done using the Statistical Package for Social Sciences (SPSS), (version 15).

## Results

**Figure 1:**
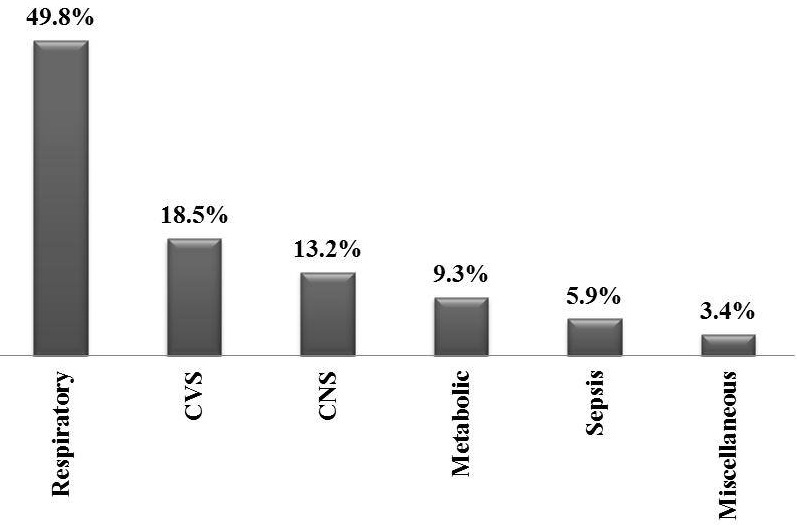
The percentage of diagnostic categories on admission to pediatric intensive care unit

During the period of the study 205 patients were admitted to the PICU. Of the 205 patients, 105(51.2%) were males and 100(48.8%) were females. The age of the patients ranged from 1 - 168 months, with a median 14 (interquartile range (IQ): 7-30) months. Patients stay in PICU ranged from 1 - 45 day with a median 6 (IQ 3 - 9) days. Respiratory illnesses such as pneumonia, bronchiolitis and status asthmaticus were the most common diagnoses among admitted patients followed by cardiovascular and neurological diseases. Figure 1 shows the percentage of diagnostic categories on admission to PICU. The predicted mortality by PIM2 score ranged from 0.03% - 96.5% with a mean 5.67% (95% CI: 3.43 - 7.91). The observed mortality rate was 8.8%, the SMR being 1.55. Table 1 shows age, gender, diagnoses on admission, DOS and PIM2 in all patients. Calibration of PIM2 using Hosmer Lemeshow goodness-of-fit-chi square test showed a good calibration of the model to studied patients in our PICU (H-L X2= 1.410 (df=8), p=0.9).

**Table 1:** Age, gender, diagnoses on admission, duration of ICU stay and pediatric index of mortality version 2 in all patients

	Survivors (n = 187)		Non-survivors (n = 18)		Total (n = 205)		P value
	N	%	N	%	N	%	
Sex							
Male	98	52.4	7	38.9	105	51.2	0.3
Female	89	47.6	11	61.1	100	48.2	
Diagnosis							
Respiratory	96	51.3	6	33.3	102	49.8	0.06
CVS	34	18.2	4	22.2	38	18.5	
CNS	21	11.2	6	33.3	27	13.2	
Metabolic	19	10.2	0	0.0	19	9.3	
Sepsis	10	5.3	2	11.1	12	5.9	
Miscellaneous	7	3.7	0	0.0	7	3.4	
Age median (IQR)	14.0 (7.0-30.0)		11.5 (2.9-27.0)		14.0 (7.0-30.0)		0.5
mean ± SD	27.7 ± 34.5		29.3 ± 42.9		27.8 ± 35.2		
DOS median (IQR)	6.0 (3.0-9.0)		5.0 (2.0-37.25)		6.0 (3.0-9.0)		0.8
mean ± SD	7.3 ± 6.4		15.6 ± 17.9		8.0 ± 8.3		
PIM2 median (IQR)	1.04 (0.41-1.75)		46.07 (1.01-73.48)		1.1 (0.4-2.2)		<0.001
mean ± SD	2.29 ± 4.99		40.77 ± 38.92		5.67 ± 16.37		

SD = standard deviation, IQR = interquartile range. DOS: Duration of stay; PMI2: pediatric index of mortality version 2

**Figure 2:**
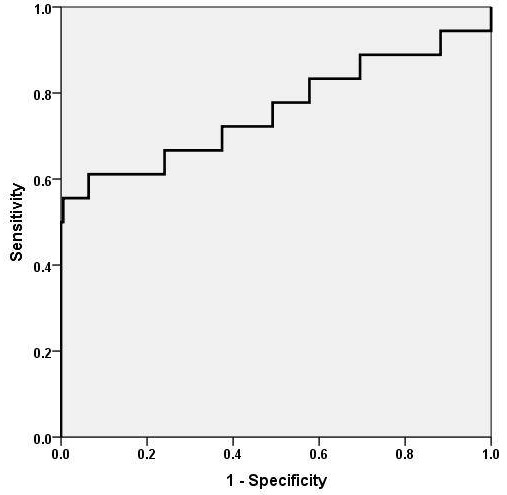
The ROC curve for pediatric index of mortality version 2

The distribution of risk deciles in the case mix is shown in Table 2. Area under the curve (AUC) of the ROC curve for PIM2 scoring system was 0.75 (95% CI: 0.60 - 0.91), P <0.001. Figure 2 shows the ROC curve for PIM2.

**Table 2:** The distribution of risk deciles in the case mix

Groups	Survivors		Non survivors		Total
	Observed (n=187)	Expected	Observed (n=18)	Expected	
1	20	20.262	1	0.738	21
2	20	20.252	1	0.748	21
3	20	20.241	1	0.759	21
4	20	20.221	1	0.779	21
5	20	20.207	1	0.793	21
6	20	20.192	1	0.808	21
7	21	20.172	0	0.828	21
8	20	20.100	1	0.900	21
9	20	19.711	1	1.289	21
10	6	5.643	10	10.357	16

Hosmer Lemeshow goodness-of-fit test for deciles of mortality risk (H-L X^2^= 1.410 (df =8), p=0.9)

## Discussion

Improvement of pediatric critical care service can be achieved by strict quality control to identify groups at high risk of death and provide adequate treatment with rational use of resources [7].

The main finding of the present study is good performance of PIM2 scoring system in Fayoum University hospital PICU.

Several studies comparing different prognostic models such as PIM, PIM2, PRISM and PRISMIII scoring systems reported good performance of PIM scoring system [5, 8, 9]. Therefore the updated version of PIM scoring system was used in our unit.

Performance of PIM2 was evaluated by assessing discrimination and calibration. Discrimination estimates the probability of concordance between outcomes and predictions. It is the ability of the model to categorize patients into two outcome groups such as survivors and non-survivors. It is assessed by measuring area under the Receiver Operating Characteristics Curve [10].

Acceptable discrimination is represented by an area under the curve of >0.7, >0.8 is good and >0.9 is excellent [11]. Several studies reported the AUC for PIM2, in our study AUC was 0.75. In other developing countries such as India [6] and Barbados [11] it was 0.81, while in Trinidad it was 0.62 [12]. In developed countries AUC 0.9 was reported from Australia and New Zealand [13], and 0.87 from Spain [14].

Calibration of a model measures the correlation between the predicted outcomes and actual outcomes over the entire range of risk prediction. Calibration was assessed by Hosmer and Lemeshow goodmess of fit test [15]. A good calibration is represented by p value >0.1 [16]. Some studies suggested modification of the coefficient of regression equation separately in each country to obtain satisfactory calibration [17, 18], while others did not support this suggestion, as this will oppose the purpose of the model [19] and validation of the PIM2 score in their study had good calibration and discrimination [20].

Calibration of PIM2 score was reported from different regions of the world. A study from Italy applied PIM2 and found that it calibrate well (X2=4.92, df=8, p=0.26) [20]. Nether Land calibrated the model and found to be acceptable (X2=4.92, df=8, p=0.77) [5]. Also in Spain, the model calibrated well (X2=4.87, df=8, p=0.85) [14]. Studies from developing countries also reported good calibration of PIM2 score, such as a study from Trinidad (X2=5.61, df=8, p=0.69) [12], and a study from Barbados (X2=5.64, df=7, p=0.58) [11]. In our study PIM2 calibrated well to the case mix of Fayoum, Egypt.

The SMR is valid measure that compare risk adjusted mortality, between different centers, but it may vary according to the case mix and care offered to the patients. Also it can be used to compare different scoring systems applied to the same setting. The SMR was reported to be 0.97 in a study from Australia and New Zealand [13]. Studies from developing countries reported varying SMRs. Studies from Trinidad and Barbados, Caribbean countries reported similar SMRs of 0.86 [11, 12], while a study from India reported SMR 1.57 [6]. The SMR of the present study is similar to the SMR reported from India. Although this high SMR may reflect under prediction of mortality by PIM2 score, it should raise the attention of medical care offered to the patients in our developing countries including the primary management in the emergency department, avoidance of delayed referral to PICU from ward and more employment of resources that could upgrade the critical care services.

Outcome evaluation of the critically ill patients is a challenging task that needs plenty of future research work [21]. Quality assessment of an ICU using severity of illness scoring system is controversial as the performance of a unit is multidimensional [22, 23].

## Conclusion

PIM2 scoring system show adequate discriminatory function and well calibrated for the case mix of patients in PICU of Fayoum, Egypt. It can be used as beneficial tool for evaluation of risk adjusted mortality. Despite this good performance of PIM2 scoring system in Fayoum University PICU, further larger scale studies in cooperation with other universities of Egypt as well as neighboring countries are required for the optimal use of the score within our region.
